# Fast Healthcare Interoperability Resources–Based Support System for Predicting Delivery Type: Model Development and Evaluation Study

**DOI:** 10.2196/54109

**Published:** 2024-04-08

**Authors:** João Coutinho-Almeida, Alexandrina Cardoso, Ricardo Cruz-Correia, Pedro Pereira-Rodrigues

**Affiliations:** 1 Faculty of Medicine, University of Porto Porto Portugal; 2 Centre for Health Technologies and Services Research University of Porto Porto Portugal; 3 Health Data Science Faculty of Medicine, University of Porto Porto Portugal; 4 Nursing School of Porto Porto Portugal

**Keywords:** obstetrics, machine-learning, clinical decision support, interoperability, interoperable, obstetric, cesarean delivery, cesarean, cesarean deliveries, decision support, pregnant, pregnancy, maternal, algorithm, algorithms, simulation, simulations

## Abstract

**Background:**

The escalating prevalence of cesarean delivery globally poses significant health impacts on mothers and newborns. Despite this trend, the underlying reasons for increased cesarean delivery rates, which have risen to 36.3% in Portugal as of 2020, remain unclear. This study delves into these issues within the Portuguese health care context, where national efforts are underway to reduce cesarean delivery occurrences.

**Objective:**

This paper aims to introduce a machine learning, algorithm-based support system designed to assist clinical teams in identifying potentially unnecessary cesarean deliveries. Key objectives include developing clinical decision support systems for cesarean deliveries using interoperability standards, identifying predictive factors influencing delivery type, assessing the economic impact of implementing this tool, and comparing system outputs with clinicians’ decisions.

**Methods:**

This study used retrospective data collected from 9 public Portuguese hospitals, encompassing maternal and fetal data and delivery methods from 2019 to 2020. We used various machine learning algorithms for model development, with light gradient-boosting machine (LightGBM) selected for deployment due to its efficiency. The model’s performance was compared with clinician assessments through questionnaires. Additionally, an economic simulation was conducted to evaluate the financial impact on Portuguese public hospitals.

**Results:**

The deployed model, based on LightGBM, achieved an area under the receiver operating characteristic curve of 88%. In the trial deployment phase at a single hospital, 3.8% (123/3231) of cases triggered alarms for potentially unnecessary cesarean deliveries. Financial simulation results indicated potential benefits for 30% (15/48) of Portuguese public hospitals with the implementation of our tool. However, this study acknowledges biases in the model, such as combining different vaginal delivery types and focusing on potentially unwarranted cesarean deliveries.

**Conclusions:**

This study presents a promising system capable of identifying potentially incorrect cesarean delivery decisions, with potentially positive implications for medical practice and health care economics. However, it also highlights the challenges and considerations necessary for real-world application, including further evaluation of clinical decision-making impacts and understanding the diverse reasons behind delivery type choices. This study underscores the need for careful implementation and further robust analysis to realize the full potential and real-world applicability of such clinical support systems.

## Introduction

### Background

The ability to provide care to both women and newborns during delivery is one of the most important aspects of health care and is often used as a metric to assess health care across different countries. Cesarean delivery is one of the most important aspects of delivering babies since it has a considerable impact on the mother’s health and well-being. Despite the increased prevalence of this procedure over the last few years, the reasons behind this trend still remain unclear. Reports suggest that this increment is a global phenomenon, with the rate of cesarean deliveries almost tripling from 6.7% to 19.1% between 1990 and 2014 [1,2]. Research on the impacts of cesarean deliveries has focused on the risk of infection, hemorrhage, organ injury, and complications related to anesthesia or blood transfusion [3,4]. There is also a higher risk of complications in subsequent pregnancies, such as uterine rupture, abnormal placental implantation, and the need for hysterectomy [5,6]. As for the infant, cesarean deliveries can lead to respiratory problems, asthma, and childhood obesity [5]. In light of this, in 2015, the World Health Organization stated that cesarean delivery rates higher than 10% were not associated with a reduction in maternal or newborn mortality, even though other complications could not be fully assessed [7]. In contrast, there is no evidence of the benefits of this procedure for women or babies when there is no clear medical need; therefore, it is paramount to focus on identifying and reducing such cases [2]. It was estimated that in 2018, there were 8.8 million unnecessary cesarean deliveries [8]. It was with this in mind that a committee was established in Portugal with the specific purpose of decreasing the percentage of cesarean deliveries nationwide. One of the policies resulting from this committee’s work was the reduction of government funding per inpatient cesarean delivery episode for hospitals with rates of cesarean deliveries above 25%; as of 2020, the number of cesarean deliveries in Portugal stands at approximately 36.3%, nearing the all-time high of 36.9% in 2009 [9]. Furthermore, studies have shown that several countries could benefit from similar policies [8]. A quantitative analysis estimated that a reduction in cesarean deliveries could save millions of dollars [10] worldwide. Therefore, lowering the proportion of cesarean deliveries can yield health and financial benefits for both institutions and patients alike. With these considerations in mind, we developed a machine learning, algorithm-based support system to assist clinical teams in identifying cases of potentially unnecessary cesarean deliveries. As such, in this paper, we propose to (1) elaborate on how clinical decision support systems for cesarean deliveries can be developed using interoperability standards; (2) understand, based on the data collected, which features have the most significant impact on predicting delivery type; (3) conduct a concise economic analysis to assess the potential financial impact of implementing the proposed clinical decision support tool; and (4) compare the system’s output with clinicians’ responses.

### Rationale and Related Work

Regarding the related work, several teams already tackled the potential of predicting the delivery type before birth. We found studies related to predicting a successful vaginal birth after a previous cesarean delivery, such as the work of Lipschuetz et al [11], where a gradient boosting method was used to predict such an event using prenatal data to do so. Grobman et al [12] performed a similar study with a multivariable logistic regression model. Different modalities of data were also used to predict delivery type. Fergus et al [13] introduced a method of predicting delivery type using fetal heart rate signals. Similarly, the work from Saleem et al [14] proposed a method for predicting delivery type using interactions between the fetal heart rate and maternal uterine contraction. Finally, some studies focus on predicting the delivery mode, such as the work of Ullah et al [15], where a boosting algorithm was used to predict a delivery mode with enriched data sets. In addition, Gimovsky et al [16] introduced decision trees to predict cesarean deliveries by physician group with an area under the receiver operating characteristic curve (AUROC) of 0.73. The works of Rossi et al [17] resulted in a 7-variable model with an AUROC of 0.78, and the works of Guedalia et al [18] resulted in a model with an AUROC of 0.82, reaching 0.93 with a first cervical examination. Finally, the works of Meyer et al [19] focused on selecting something suitable for a trial of labor after cesarean delivery with an area under the precision recall curve graph around 0.351. However, to the best of our knowledge, there was no model tested in clinical practice with an interoperable format of communication such as Fast Healthcare Interoperability Resources (FHIR), which tried to not only predict delivery type but also provide support about possibly wrong deliveries, and none with simulation about financial implication, making our paper a potential novelty on different dimensions.

## Methods

### Materials

Data were retrospectively collected from 9 different public Portuguese hospitals across the country, focusing on obstetric information and encompassing maternal data, various fetal data points, and the method of delivery retrospectively. The inclusion criteria are all mothers with a registered outcome of the pregnancy from 2019 to 2020. There were no exclusion criteria. Each institution used identical electronic health record software, ensuring that the data columns remained consistent.

### Clinical Comparison

The clinical comparison was performed by sending questionnaires to clinicians with a relationship with obstetrics to assess 10 patients, with only access to the variables used by the model, and to answer 3 questions for each. The first question was to give a score from 1 to 10 of how likely it was that a patient would give birth through cesarean delivery, the second question was to select the feature or variable that most influenced the decision, and the third question was to select which feature they would require to make a better assessment. We sent the questionnaire to 20 people and obtained 6 answers, totaling 60 patient assessments. For these 10 patients, we also predicted the delivery type using our model to compare it with the clinicians’ answers. These patients were new and were not seen by the model during the training phase.

### Analysis

All null representations were standardized. Data were prepossessed by removing features with high missing rates (>90% overall). The imputation process was performed using the k-nearest neighbor imputation method (for continuous variables) or a new category (NULLIMP) for categorical variables. Weight was categorized into percentiles defined specifically for Portuguese babies [20]. For this study, the birth type was reduced to binary. All assisted birth were merged into vaginal birth, and cesarean delivery remained as the other class. Procedures and diagnoses were also used and were encoded as binary features, and we took the time to analyze each one of them to avoid leakage because there were procedures obviously related to cesarean deliveries and vaginal deliveries. Feature creation was performed through the free-text variable related to the prescribed medication. Medicine names were collected from it and converted into Anatomical Therapeutic Chemical classification group level 4, which represents chemical subgroups. We also created some new features from data in the data set, namely new categories related to the labor and condition of the baby. In addition, data quality issues were addressed, such as impossible values that were transformed into null values. The main variables affected by data quality were BMI or weight and gestational age. The data were split into training and test sets in a 0.75:0.25 manner. From the overall data sets that comprised over 200 columns, only a few columns were selected (see Table 1). We used a mixture of features selected by surveying the literature [21-23] and features with a high correlation with the outcome. The tested models were logistic regression, decision tree, random forest, 3 different boosting methods (as implemented by extreme gradient boosting [XGBoost], light gradient-boosting machine [LightGBM], and scikit learn), and a linear model based on stochastic gradient descent.

**Table 1:** Distribution of features used for prediction (N=73,351).

Variable	Value	Mode^a^
Mother’s age (y), mean (SD)^b^	31.0 (5.6)	N/A^c^
Weight prepregnancy (kg), mean (SD)	65.8 (13.9)	N/A
Weight on admission (kg), mean (SD)	78.6 (14.2)	N/A
BMI (kg/m^2^), mean (SD)	25.0 (5.4)	N/A
Previous eutocic delivery, mean (SD)	0.4 (0.7)	N/A
Previous vacuum-assisted delivery, mean (SD)	0.1 (0.3)	N/A
Previous forceps, mean (SD)	0.0 (0.1)	N/A
Previous cesarean delivery, mean (SD)	0.1 (0.4)	N/A
Fetal presentation on admission, n (%)^d^	19,305 (26.32)	Cephalic
Bishop score, mean (SD)	5.5 (3.0)	N/A
Gestational age on admission (mo), mean (SD)	38.9 (1.9)	N/A
Premature rupture of the membrane, n (%)	64,541 (87.99)	No
Chronic hypertension, n (%)	71,649 (97.68)	No
Gestational hypertension, n (%)	71,700 (97.75)	No
Preeclampsia, n (%)	72,104 (98.3)	No
Gestational diabetes, n (%)	65,876 (89.81)	No
Gestational diabetes treated with a diet, n (%)	69,162 (94.29)	No
Gestational diabetes treated with insulin, n (%)	71,942 (98.08)	No
Gestational diabetes treated with oral antidiabetic drugs, n (%)	71,737 (97.8)	No
Maternal diabetes, n (%)	72,991 (99.51)	No
Type 2 diabetes, n (%)	73,233 (99.84)	No
Presentation at birth, n (%)	68,950 (94)	Vertex presentation
Delivery, n (%)	39,507 (53.86)	Spontaneous
Gestational age on birth (mo), mean (SD)	39.0 (1.8)	N/A
Smoking during pregnancy, n (%)	64,871 (88.44)	No
Alcohol consumption during pregnancy, n (%)	72,360 (98.65)	No
Consumed drugs during pregnancy, n (%)	73,226 (99.83)	No
Number of pregnancies (with current), mean (SD)	1.9 (1.1)	N/A
Pregnancy type, n (%)	62,656 (85.42)	Spontaneous
Surveillance, n (%)	71,664 (97.7)	Yes
Hospital surveillance, n (%)	49,739 (67.81)	Yes
Pelvis adequacy, n (%)	12,844 (17.51)	Adequate
Consistency of the cervix, mean (SD)	1.6 (0.6)	N/A
Fetal station, mean (SD)	0.8 (0.8)	N/A
Dilation of the cervix, mean (SD)	1.3 (0.8)	N/A
Effacement of the cervix, mean (SD)	1.2 (1.2)	N/A
Position of the cervix, mean (SD)	0.6 (0.7)	N/A
Hematologic disease, n (%)	70,182 (95.68)	No
Respiratory disease, n (%)	70,131 (95.61)	No
Cerebral disease, n (%)	72,470 (98.8)	No
Cardiac disease, n (%)	68,194 (92.97)	No
Neuroaxis techniques, n (%)	50,978 (69.5)	1
Number of children, mean (SD)	0.6 (0.8)	N/A

^a^Mode, number, and percentage for categorical variables.

^b^Mean and SD for continuous variables.

^c^N/A: not applicable.

^d^

The evaluation was performed with repeated stratified cross-validation with 10 splits and 2 repetitions, with 2 full cycles of dividing the training set into 10 equal parts and using 9 as the training set and 1 as the validation set. The results are shown in Table 2. The application programming interface (API) for serving the prediction model was developed using FastAPI. We wrote all the code in Python (version 3.9.7; Python Software Foundation).

**Table 2:** Repeated cross-validation (10 × 2) results in the training set with mean AUROC^a^ and 95% CI for the best hyper-parameters found for each algorithm. Wilcoxon test was used for comparing with the best performing algorithm.

Algorithm	AUROC (95% CI)	*P* value
XGBoost^b^	0.8809 (0.88-0.8818)	—^c^
Decision tree	0.8338 (0.8328-0.8347)	≤.001
Logistic regression	0.8716 (0.8708-0.8724)	≤.001
AdaBoost^d^	0.8753 (0.8744-0.8763)	≤.001
LightGBM^e^	0.8805 (0.8795-0.8815)	.003
Stochastic gradient descent	0.8704 (0.8695-0.8712)	≤.001
Random forest	0.8752 (0.8744-0.8761)	≤.001

^a^AUROC: area under the receiver operating characteristic curve.

^b^XGBoost: extreme gradient boosting.

^c^Not available.

^d^AdaBoost: Adaptive Boosting.

^e^LightGBM: light gradient-boosting machine.

### Ethical Considerations

This study received institutional review board approval from all hospitals included in this study with the following references: Centro Hospitalar São João (08/2021), Centro Hospitalar Baixo Vouga (12-03-2021), Unidade Local de Saúde de Matosinho (39/CES/JAS), Hospital da Senhora da Oliveira (85/2020), Centro Hosptilar Tamega Sousa (43/2020), Centro Hospitalar Vila Nova de Gaia/Espinho (192/2020), Centro Hospitalar entre Douro e Vouga (CA-371/2020-0t_MP/CC), and Unidade Local de Saúde do Alto Minho (11/2021). All methods were carried out per relevant guidelines and regulations. The need for informed consent was waived by the ethics committee.

## Results

### Descriptive Statistics

The number of samples varied across the hospitals, ranging from 2364 to 18,177. Distributions of the selected variables are presented in Table 1. The sum of all samples was 73,351. The outcome variable distribution is stated in Table 3.

**Table 3:** Distribution of delivery methods.

Type of delivery	Value (n=73,351), n (%)
Cesarean delivery	19,803 (27)
Vaginal	38,189 (52)
Instrumental delivery	15,359 (21)

### Model

The AUROC is presented in Table 2 for the best hyper-parameters found for each algorithm in the training data. All models used the variables indicated in Table 1.

While XGBoost was the best-performing algorithm, we selected LightGBM [24] because of its speed and lower memory requirements, which we believe are better suited for deployment in a low-hardware environment. The threshold selected for deploying the model was 0.7457, which rendered the metrics in the test set, as shown in Table 4.

**Table 4:** Performance metrics in the test set with chosen threshold.

Metric	Value
Accuracy	0.8052
Sensitivity	0.8223
Precision	0.9023
*F*_1_ score	0.8605

### Deployment

**Figure 1:**
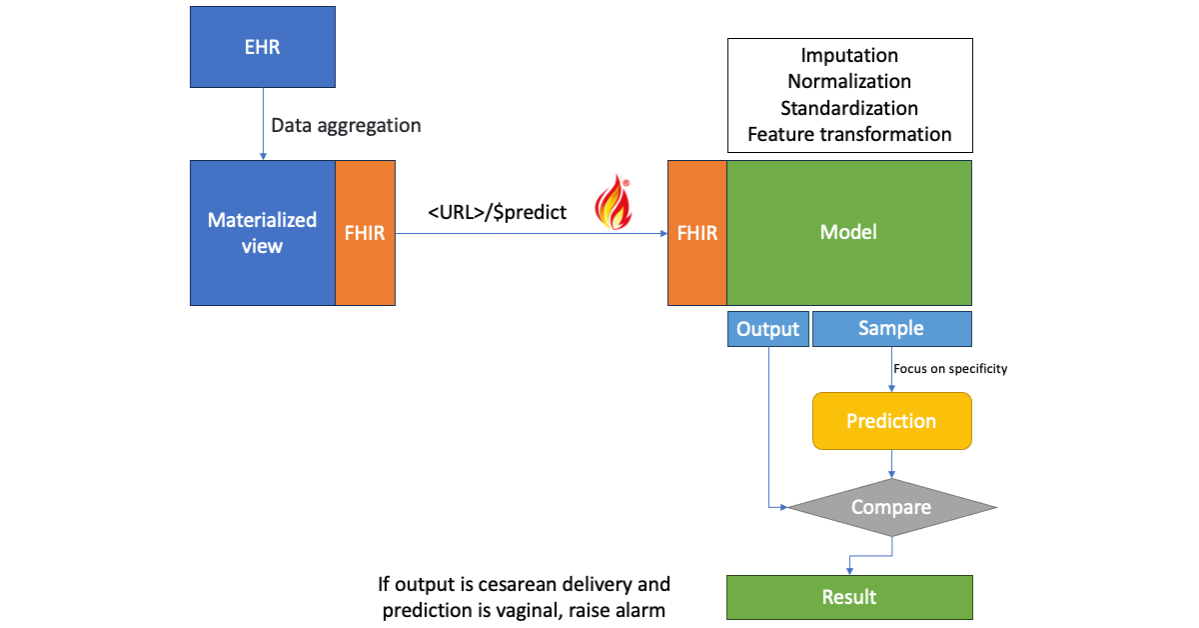
Deployment and decision mechanism of the model. EHR: electronic health record; FHIR: Fast Healthcare Interoperability Resources.

The purpose of this model is to serve as an API for usage within a health care institution and to act as a supplementary clinical decision support tool for obstetrics teams. For this to happen, a health information system must make the requests to the API. Even though a concrete, vendor-specific information model and input health information system were used, we hope to create a more interoperable clinical decision support system that can be used by every system that acts on birth and obstetrics departments. Therefore, we built it around the HL7 FHIR standard (R5 version) to simplify the method of interacting with the API. This decision, opposed as to using a proprietary model for the data, sits upon the usage of FHIR resources: bundle and observation for request and returning the result as a message through a custom operation called “$predict.” It is intended to publish the profiles of these objects to facilitate access to the API using standardized mechanisms and data models. The current build of the profiles can be seen in the published FHIR implementation guide where the current specifications are described in detail [25]. The process is illustrated in Figure 1. We deployed this model in production in a single hospital without a user interface, collecting only the data and predictions for later discussion and analysis. We collected 3231 requests. During this period, 123 (3.8%) alarms were triggered. From this, we tried to understand the level of certainty for the decision and check the difference from the threshold of these alarms. The distance to the threshold for 73 was lower than 0.1 and was bigger than 0.1 for 50 (1.55%) cases.

### Clinical Comparison

**Figure 2:**
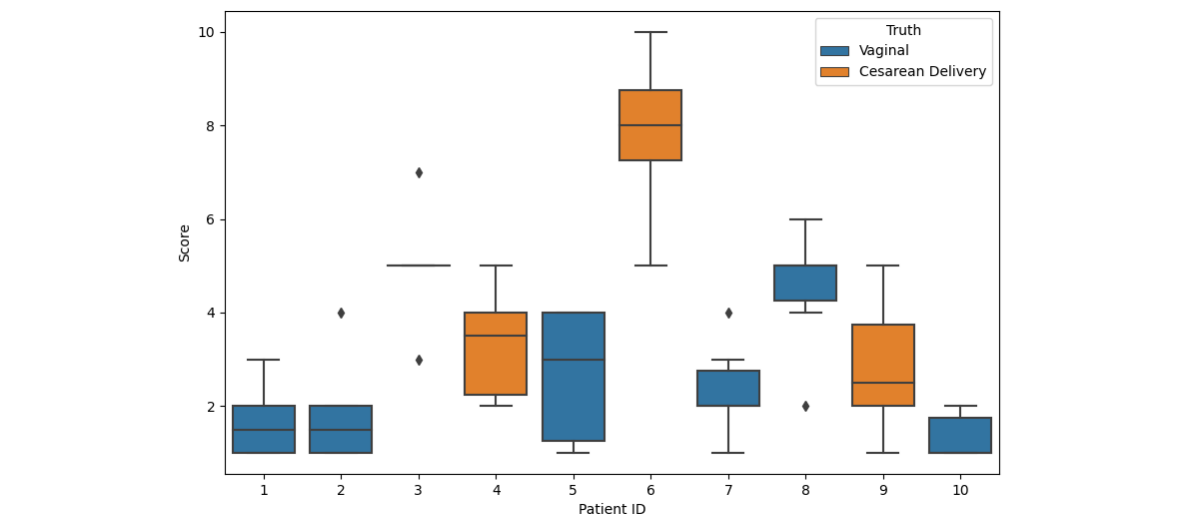
Validation data. The color represents the actual birth type. The boxplot represents the median and IQR of the reviewers, and the x-axis represent the patient cases. There were 6 vaginal births and 4 cesarean deliveries. ID 4 represents wrong predictions of the model.

The median scores given by each clinician are presented in Figure 2. We also predicted the result using our model as stated in Figure 2. The model misclassified only 1 record (ID 4). As for the analysis of missing features for the responders, they were divided into 3 categories: (1) existent in the data set but not included in the model, (2) nonexistent in the data set, and (3) existent in the data set and included but that particular information was not filled for the patient assessed. Out of 60 responders, this rendered a total of 37 (62%) with nonexistent features and 23 (38%) with existent features but no information was provided at that moment. No feature mentioned existed in the data set but had not been included in the model. From the 37 nonexistent features, 14 (38%) were new clinical assessments, 14 (38%) were linked to information from previous births, 5 (15%) were connected to more in-depth information about provided information (ie, a motive for induction), and 4 (11%) were related to the mother’s choice (if she wanted a cesarean delivery). As for feature importance, from the 60 answers, 33 (55%) stated that labor was the most important factor. Further, 9 (15%) stated the number of previous vaginal births, 5 (8%) stated the evolution of weight, and another 5 (8%) stated the number of previous cesarean deliveries as being the most important. The remaining 8 (14%) were various features, such as BMI, neuroaxis techniques, gestational age, and weight of the mother. Of all of these, 54 (90%) were in the top 10 features of the model.

### Potential Financial Impact

The financial support provided to public hospitals in Portugal is partially tied to the rate of cesarean deliveries. To assess the potential impact of this mechanism on Portuguese public hospitals, we conducted a simulation. We got data for every public hospital for the last 12 months and applied a 3.8% reduction (the rate of warnings triggered in the new data set) and recalculated the rate of cesarean deliveries. The increase in support was calculated by the state-mandated rate as shown in Table 5. With this new rate, we observed that implementing our tool would result in financial benefits for 30% (15/48) of the public hospitals. Specifically, 5 hospitals would begin receiving support instead of no support at all. Further, 3 hospitals would experience a doubling of their financial benefit, while 2 hospitals would see a 50% increase. Furthermore, 1 hospital would receive an additional one-third of financial support. If we assumed that only half of the warnings found in the new data were actually true (1.9%), we found that only 6 hospitals would be benefited: 3 from 0 to 0.25, 2 from 0.25 to 0.50, and 1 from 0.50 to 0.75.

**Table 5:** The rule set for state-provided financial support indexed to cesarean deliveries. x is the current payment of a cesarean delivery inpatient episode [26].

Rate of cesarean deliveries	Support
<25%	x
25%-26.4%	0.75x
26.5%-27.9%	0.5x
28%-29.4%	0.25x
>29.5%	0

## Discussion

### Principal Findings

The first thing to address about this model is the number of biases that we introduced in the model by choice. We joined all vaginal delivery types into a single category (assisted and nonassisted), which introduces a bias since these delivery modes are indeed different. Second, the fact that we want to predict if the delivery type was wrongly chosen, mainly for the case of a cesarean delivery that did not need to be so, is also a bias. We used this approach because the initially collected data did not have the representation of such events. Thus, the biases of possibly wrong delivery types were present in the training data. We attempted to minimize this issue by selecting a threshold that gave the model higher sensitivity than specificity so that only large probabilities would trigger an alarm for human consideration. Parallel to this, we are starting to gather labeled cases, with the help of clinicians to create a better training data set. Furthermore, since the data were collected from different hospitals, differences in the data input can also occur. Even though the health information system is the same, the processes that originate the data and are being used for secondary purposes could introduce several biases in the data. This is an issue that was accepted from the start regarding the mechanism of data collection and model training. Despite this, we reached a model with a very high AUROC (88%, 95% CI 0.8795-0.8815), which is encouraging when compared to the state-of-the-art, which is between 0.73 and 0.82 [16-18]. Moreover, assuming that more data are provided, and proper labeling is done regarding the outcome variable (such as a clinical evaluation of needless cesarean deliveries) is added as well, a better model could be developed.

Regarding the preliminary clinical evaluation, it was only possible to obtain an overview of the possible comparison due to the number of responders. Despite that, the results are encouraging, since the model seems to behave better than humans with the data provided. However, this is a biased vision since clinicians in the real world have access to more data and information than the model has. It is encouraging, but caution is advised before more tests and evaluations are done. As for the deployment, future work could be the improvement of the API to map all variables to an ontology such as SNOMED CT or similar, making it easier for every system and person to access it and obtain a suggestion of the delivery type. Finally, we believe the assessment can be improved. A more robust clinical assessment is necessary, as well as a thorough analysis of the impact of the tool in the real world, since we need to create the bridge between the results of the model and how clinical decisions are affected by it. A full cost-effectiveness analysis is also necessary to understand the real-world impact of the model. Further, 1 interesting result is the fact that 38% of the answers regarding the most important data element missing from the patient record refer to data that are being collected but was missing for that specific patient, raising an important question about data input methodology, interoperability, and quality. If we cannot have access to data when these matter the most, these can become meaningless. Missing data are a problem of biomedical data as a whole. However, when specifically targeted at machine learning usage of this data for predicting something, we did not find any work comparing them with clinicians. However, we did find reporting of similar missing values in obstetrics data [27] and we also found works of a similar nature using machine learning models with robust handling of missing data such as XGBoost [28] to counter this problem. This indicates that our model has the potential to counter the missing data problem as well since LightGBM can also handle missing data natively.

### Conclusions

We believe we have developed a robust system capable of detecting potentially incorrect cesarean delivery decisions, which could positively impact real-world medical practice. However, before implementation, several challenges must be addressed, particularly the need for further evaluation of the system’s impact on clinical decision-making and the reasons underlying suboptimal delivery-type decisions. Cesarean deliveries may be performed for various reasons, from a mother’s preference to a decision made by the obstetrics team. This system is not designed to impede medical practice or to highlight flawed decisions, potentially scrutinizing specific professionals. Such caution is necessary when implementing systems like these. While having a high AUROC is beneficial, the real-world impact is another consideration. The assumptions and biases associated with autonomous systems supporting clinical practice must be carefully considered. Nonetheless, the metrics and results we have achieved so far are promising for positively influencing health and economic outcomes.

## Abbreviations

API: application programming interface
AUROC: area under the receiver operating characteristic curve
FHIR: Fast Healthcare Interoperability Resources
LightGBM: light gradient-boosting machine
XGBoost: extreme gradient boosting

## Data Availability

The data cannot be publicly available due to privacy and ethical restrictions. The code is available [29].
